# Identification of immune biomarkers in recent active pulmonary tuberculosis

**DOI:** 10.1038/s41598-023-38372-7

**Published:** 2023-07-17

**Authors:** Sobia Naz Shaukat, Eliseo Eugenin, Faizan Nasir, Rafiq Khanani, Shahana Urooj Kazmi

**Affiliations:** 1 Immunology and Infectious Diseases Research Laboratory (IIDRL), Department of Microbiology, Karachi University, Karachi, Pakistan; 2 Department of Neurobiology, University of Texas Medical Branch (UTMB), Galveston, TX USA; 3 Department of Immunology, Dadabhoy Institute of Higher Education, Karachi, Pakistan; 4 Dow University of Health Sciences, Ojha Campus, Karachi, Pakistan; 5 Department of Biological and Biomedical Sciences, Aga Khan University Hospital, Stadium Road, P.O. Box 3500, Karachi, 74800 Pakistan

**Keywords:** Immunology, Microbiology, Molecular biology, Biomarkers

## Abstract

Tuberculosis (*TB*) has remained an unsolved problem and a major public health issue, particularly in developing countries. Pakistan is one of the countries with the highest tuberculosis infection rates globally*. However, methods or biomarkers to detect early signs of TB infection are limited. Here,* we characterized the mRNA profiles of immune responses in unstimulated *Peripheral blood mononuclear cells* *obtained from* treatment naïve patients *with early signs* of active pulmonary tuberculosis without previous history of clinical TB. We identified a unique mRNA profile in active TB compared to uninfected controls, including cytokines such as IL-27, IL-15, IL-2RA, IL-24, and TGFβ, transcription factors such as STAT1 and NFATC1 and immune markers/receptors such as TLR4, IRF1, CD80, CD28, and PTGDR2 from an overall 84 different transcripts analyzed. Among 12 significant differentially expressed transcripts, we identified five gene signatures which included three upregulated IL-27, STAT1, TLR4 and two downregulated IL-24 *and* CD80 that best discriminate between active pulmonary TB and uninfected controls with AUC ranging from 0.9 to 1. Our data identified a molecular immune signature associated with the *early stages of active pulmonary tuberculosis and it could be further investigated as a potential biomarker of pulmonary TB.*

## Introduction

Tuberculosis (TB) is a serious global public health challenge due to its associated disabilities, deaths, contagious nature, long incubation period, insidious onset leading to delayed diagnosis and severe long-term consequences^1,2^. Although TB is curable, statistics in several countries, including Pakistan, remain alarming. According to the WHO TB Report 2021, an estimated *1.6* million people died due to TB, while *10.6* million new TB cases occurred globally in *2021*^3^, including *6* million men, *3.4* million women and *1.2* million children^1–4^. Pakistan stands sixth among 30 high-burden countries for TB with an incidence rate of *264/100,000* compared to *3/100, 000* in the US, according to the World Bank report *2021*^5^.

*Mycobacterium tuberculosis* (Mtb) is the main causative agent of TB. Despite significant advances in identifying Mtb cases, treatment and eradication, there is a significant lack of information about the initial immune responses against Mtb in humans^2,6^. *It is known and well-reported that adaptive immunity plays an important* role in TB pathogenesis^7,8^. During the early stages of Mtb infection, cell-mediated immune responses are required to localize Mtb into a particular lung area which is mainly associated with the expression of cytokines/chemokines essential for the recruitment of inflammatory cells into the site of infection, including IL-1β, IL-2, IL-12, IFNγ and TNFα^9–11^. In contrast, cytokines associated with an anti-inflammatory profile, including IL-4, IL-13, and IL-10, counteract the pro-inflammatory responses and help Mtb to evade the protective immune responses and establish an active infection^12^. *Therefore, the dynamics of the inflammatory responses hold significant importance in the early diagnosis and outcome of the pathogenesis.*

Several studies reported higher levels of pro-inflammatory responses in active TB^7,11,13–16^, compared to chronic TB, in which higher levels of anti-inflammatory responses were observed^13,16–18^. Most of these studies were performed on serum, plasma, or whole blood of the Mtb-infected subjects *using immunoassays*^13,19–24^*. Transcriptional studies were also conducted mainly on whole blood or stimulated PBMCs of TB patients *^25–30^*.* However, assessment of the immune markers from ex-vivo stimulated*, or cultured* PBMCs provide an accurate reflection of the onset of in-vivo immune responses is not guaranteed^31–34^.

*Only a few transcriptional studies were performed on unstimulated PBMCs. Most of these studies used cohorts with active, latent or drug-treated Mtb cases without a proper selection of early cases of infection*^35–37^*. Hence, limited* data is available on the transcriptional profiling of pro and anti-inflammatory markers from unstimulated (naïve) PBMCs of patients with an early and treatment naïve Mtb infection^38–40^*.* We aimed to analyze unstimulated (naïve) PBMCs, isolated from recently diagnosed active pulmonary TB patients *within 4 to 6 weeks of first clinical manifestation*s and transcriptionally characterized these PBMCs to study onset in-vivo immune responses during initial phase of the Mtb infection to find out some better biomarkers for the early detection of infection and immune activation.

## Results

### Characteristics of the population examined

The selected population consisted of thirty-one (31) individuals categorized as having *recent* active cases of pulmonary tuberculosis by TB hospital, as indicated in the methods. The physical examination indicated that 87% of the individuals had cough with expectoration (80%), fever (70%) and weight loss (58%). The diagnostics were confirmed by chest radiography in 90% of the cases analyzed, with no old lesions detected. The duration of symptoms was 4 to 6 weeks from the first clinical manifestations, and *all patients were recruited within this period*. Laboratory examination further revealed that all the patients had positive findings for Acid Fast Bacilli (ABF) on Zeihl-Neelsen staining with smear grading: 1 + (32%), 2 + (13%), 3 + (48%), followed by culturing and nucleic acid identification for Mycobacterium tuberculosis complex (MTBC). Demographic details showed that 39% were females with a mean age of 19.5 years, S.D. ± 3.08, while 61% were males with a mean age of 27.2 years, S.D. ± 10.4, in our study group. Among all, nearly 50% of the patients had a history of TB in the family. Similarly, the mean weight of the patients was found to be 44.9 kg with S.D. ± 10.2 (Table 1), whereas none of the patients with co-morbidity and co-infections (HIV, HCV, and HBV) were included in this study. Furthermore, the socioeconomic details of the enlisted participants demonstrated that they were from low-income families (approx. 100 USD,  ± 25 S.D.) with low dietary and literacy conditions. *A critical point of our cohort is that all individuals involved lack previous TB history, and imaging showed no old lung lesions. Thus, our population corresponds to a newly diagnosed cohort with active TB based on newly acquired symptoms and clinical testing and never had Mtb treatment or long-term infections *(Table 2)*. In addition, the present study examined twenty-five uninfected people as controls*, of which 68% were males with a mean age of 34.8, SD = 3 and 32% were females with a mean age of 26.7, SD = 2.8 (Table 1). The group was tested for HCV, HBV, HIV infection, Tuberculin Skin Test (TST) and chest X-rays to discard TB. All the participants of this group were negative for these conditions.

**Table 1:** Patient Information.

Demographics	TB group (n = 31)	Control group (n = 25)	*p* value
Gender			0.923
Male	61%	68%	
Female	39%	32%	
Age(years)			0.0016
15– < 35	80%	64%	
35–55	20%	36%	
Mean age (years)			
Female	19.5(SD ± 3.08)	26.7 (± 2.8)	
Male	27.2(SD ± 10.4)	34.8(± 3.0)	
Mean weight (Kg)	44.9 kg	66.4 kg	9.93e−9

**Table 2:** Patient Characteristics.

Clinical features of TB patients	Frequency (%)
Fever	70
Cough	87
Expectoration	80
Hemoptysis	32
Weight loss	58
Chest pain	35
Smear microscopy	
Negative	21
1 to 9 AFB in 100 fields 6%	0
10–99 AFB in 100 Fields (1+)	32
1–10 AFB in each field (2+)	13
> 10 AFB in each field (3+)	48
AFB culturing	100
Family history for TB	48
Positive chest X-rays	90
ATT	0

### Analysis of mRNA expression in PBMCs obtained from individuals with active pulmonary TB and uninfected control

**Figure 1:**
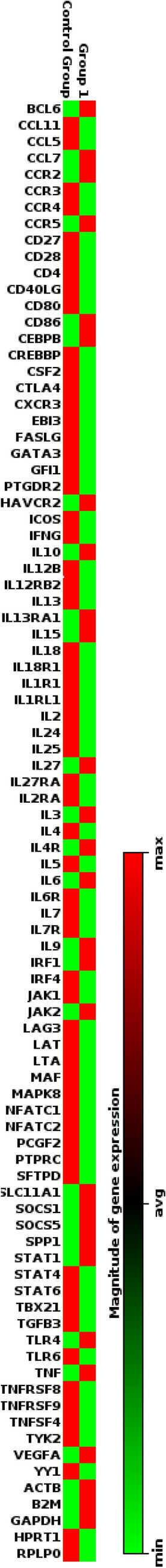
Heatmap showing differential expression of 84 genes in active pulmonary TB patients (Group 1) and uninfected controls (Control Group). The magnitude of gene expression is defined using color code (Black = average, Red = maximum, Green = minimum).

**Figure 2:**
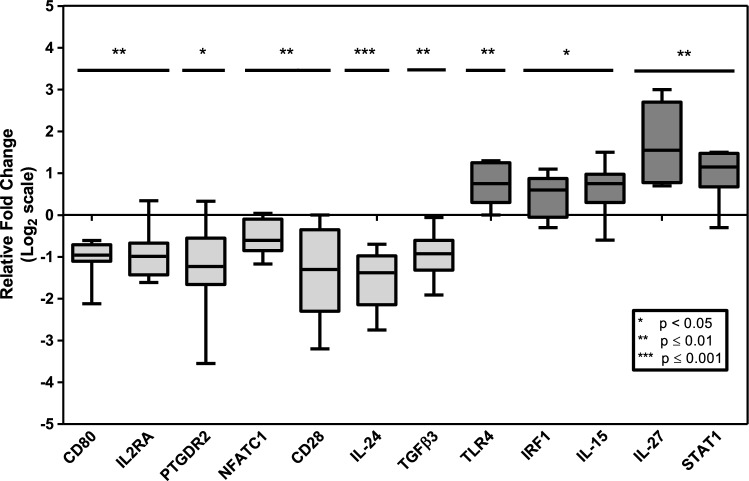
Box plot showing significant expression of 12 genes including TLR4, IRF1, IL-15, IL-27, STAT1 as upregulated genes and CD80, IL-2RA, PTGDR2, NFATC1, CD28, IL-24, TGFβ3 as downregulated genes. The plot shows whiskers from minimum (lower quartile) to maximum (upper quartile), with the middle line in each box as the median. Statistical significance was calculated using the student’s T-test with two-tailed distribution, unpaired and two-sample equal variance analysis, *p < 0.05, **p < 0.01, ***p ≤ 0.001.

**Figure 3:**
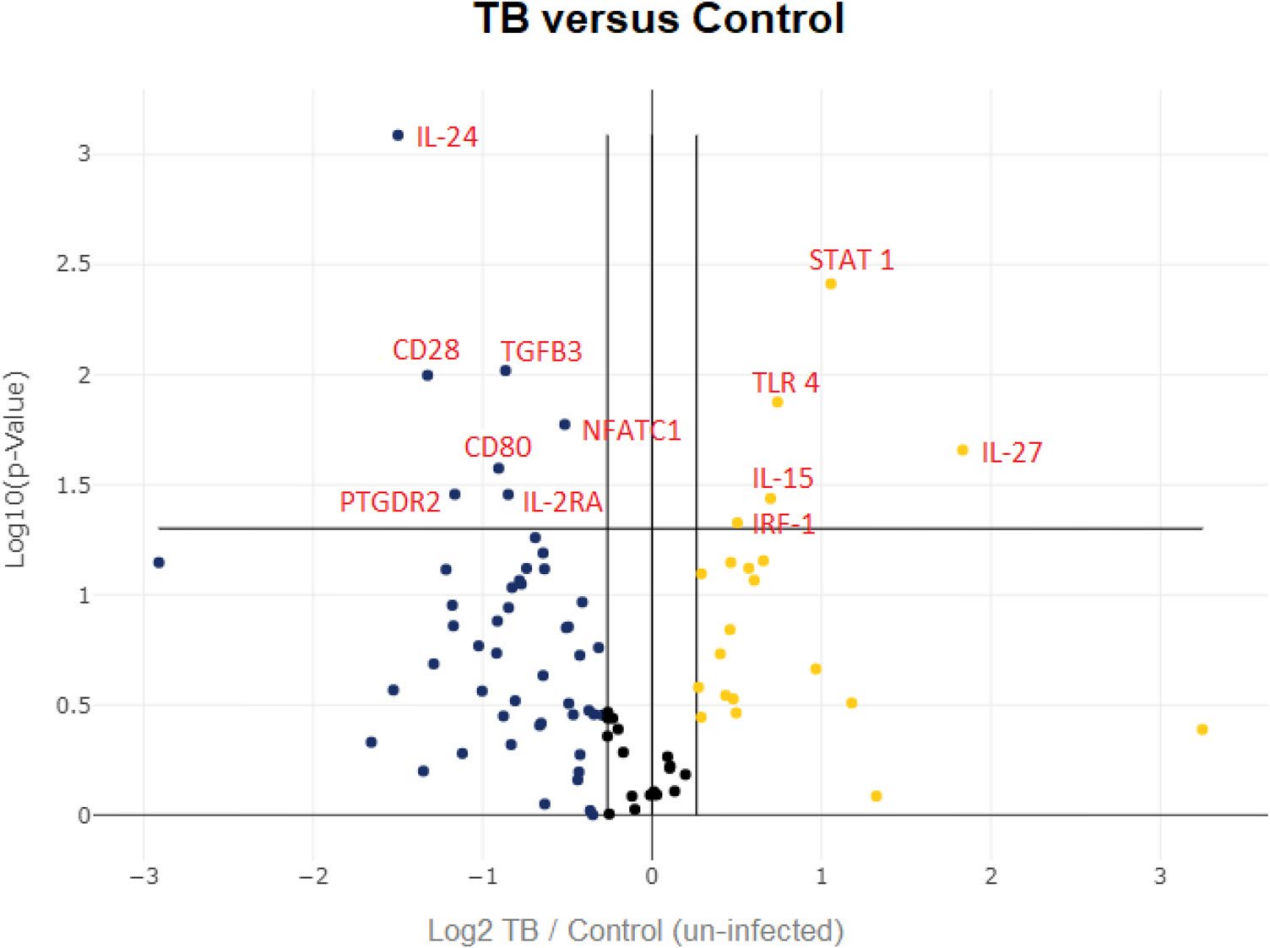
The volcano plot shows the distribution of differentially expressed genes with respect to the fold change in gene expression on the x-axis and their statistical significance on the y-axis. The middle vertical line shows genes with unchanged expression, whereas data points on the right and left side show upregulated and downregulated gene expression, respectively. Significant genes (*p* < 0.05) are indicated above the central horizontal line in red color.

We focused on unstimulated (naïve) PBMCs to identify biomarkers of an early immune response in active pulmonary TB. Thus, using PBMCs from active pulmonary TB and healthy individuals, we examine the mRNA profile expression of 84 inflammatory and 5 housekeeping genes to cluster them into different functional mRNA populations as indicated in the heat map shown in Fig. 1. Most mRNA expressed in PBMCs obtained from healthy (uninfected) and Mtb infected individuals were in the minimal to average expression area (green color). However, several clusters had high expression of specific transcripts (red clusters). We identified 12 transcripts differentially expressed between active TB versus control individuals (Table 3). Five transcripts were upregulated, including IL-15, IL-27, STAT1, TLR4, and IRF1, from 84 analyzed transcripts (Fig. 2, *p* < *0.05*). Also, we identified seven downregulated transcripts in active TB versus uninfected individuals, including IL-2RA, IL-24, TGFβ, CD28, CD80, NFATC1, and PTGDR2, as shown in Box plot (Fig. 2*, p* < *0.05*). In contrast, the statistical significance of differentially expressed transcripts between active TB and uninfected controls is shown via Volcano plot. Transcripts highlighted in red are significant (Fig. 3, *p* < 0.05).

**Table 3:** Transcripts expression profile.

Category	Genes	*P* value	Fold change (FC)	Log_2_FC	Category	Genes	*P* value	Fold change (FC)	Log_2_FC
Chemokine and their receptors	CCL11	0.2950	0.63	− 0.67	Cell surface markers	CD27	0.3831	0.64	− 0.64
	CCL5	0.0550	0.62	− 0.69		CD28*	**0.0101**	**0.4**	**− 1.32**
	CCL7	0.3096	2.26	1.18		CD4	0.6380	0.74	− 0.43
	CCR2	0.0757	1.49	0.58		CD40LG	0.1840	0.53	− 0.92
	CCR3	0.2707	0.35	− 1.51		CD80*	**0.0100**	**0.53**	**− 0.92**
	CCR4	0.9901	0.84	− 0.25		CD86	0.0700	1.58	0.66
	CCR5	0.0858	1.52	0.60	Transcription factors	NFATC1*	**0.0168**	**0.7**	**− 0.51**
	CXCR3	0.0713	0.13	− 2.94		NFATC2	0.2058	0.41	− 1.29
Cytokine and their receptors	IFNγ	0.1315	0.53	− 0.92		STAT1*	**0.0039**	**2.08**	**1.06**
	IL12B	0.1383	0.44	− 1.18		STAT4	0.0893	0.59	− 0.76
	IL12RB2	0.2324	0.64	− 0.64		STAT6	0.8219	0.92	− 0.12
	IL18	0.3630	0.83	− 0.27		TBX21	0.0861	0.58	− 0.79
	IL18R1	0.3914	0.63	− 0.67		GATA3	0.0767	0.43	− 1.22
	IL1R1	0.6926	0.74	− 0.43		CEBPB	0.1852	1.32	0.40
	IL1RL1	0.4791	0.56	− 0.84		CREBBP	0.0763	0.64	− 0.64
	IL2	0.1143	0.56	− 0.84	Signaling pathways	IRF1*	**0.0470**	**1.42**	**0.51**
	IL2RA*	**0.0170**	**0.56**	**− 0.84**		IRF4	0.4074	0.87	− 0.20
	IL10	0.2168	1.95	0.96		JAK1	0.3348	0.77	− 0.38
	IL13	0.8121	0.99	− 0.01		JAK2	0.2628	1.21	0.28
	IL13RA1	0.8094	1.02	0.03		TLR4*	**0.0081**	**1.67**	**0.74**
	IL24*	**0.0007**	**0.35**	**− 1.51**		TLR6	0.3490	0.79	− 0.34
	IL25	0.3638	0.85	− 0.23		SPP1	0.8211	2.5	1.32
	IL27RA	0.3498	0.72	− 0.47		LAT	0.5322	0.74	− 0.43
	IL27*	**0.0010**	**3.56**	**1.83**		MAPK8	0.1079	0.75	− 0.42
	IL15*	**0.0365**	**1.62**	**0.70**		TYK2	0.0758	0.6	− 0.74
	IL3	0.7800	1.1	0.14	Immune check point markers	ICOS	0.5192	0.89	− 0.17
	IL4	0.3026	0.57	− 0.81		CTLA4	0.0926	0.56	− 0.84
	IL4R	0.7837	1.01	0.01		LAG3	0.1704	0.49	− 1.03
	IL5	0.9549	0.78	− 0.36	Growth factors	CSF2	0.6319	0.39	− 1.36
	IL6	0.2967	1.39	0.48		VEGFA	0.6129	1.08	0.11
	IL6R	0.1882	0.74	− 0.43	Apoptotic markers	MAF	0.3114	0.71	− 0.49
	IL7	0.0646	0.64	− 0.64		BCL6	0.2864	1.35	0.43
	IL7R	0.9979	0.78	− 0.36		PCGF2	0.8918	0.64	− 0.64
	IL9	0.3435	1.41	0.50		FASLG	0.1115	0.44	− 1.18
Immune suppressive markers	GFI1	0.2735	0.5	− 1.00		LTA	0.4669	0.32	− 1.64
	TGFβ*	**0.0096**	**0.55**	**− 0.86**	Other Immune transcripts and receptors	SFTPD	0.3554	0.54	− 0.89
	SOCS1	0.1438	1.38	0.46		SLC11A1	0.0712	1.38	0.46
	SOCS5	0.6005	1.08	0.11		HAVCR2	0.3594	1.22	0.29
	PTPRC	0.1737	0.8	− 0.32					
TNF family	TNF	0.6553	1.15	0.20		YY1	0.3412	0.83	− 0.27
	TNFRSF8	0.4387	0.83	− 0.27		EBI3	0.5254	0.46	− 1.12
	TNFRSF9	0.1410	0.7	− 0.51		PTGDR2*	**0.0349**	**0.45**	**− 1.15**
	TNFSF4	0.1397	0.71	− 0.49					

Significant values are indicated in bold.

### Newly active pulmonary TB individuals have a strong and specific pro-inflammatory response

**Figure 4:**
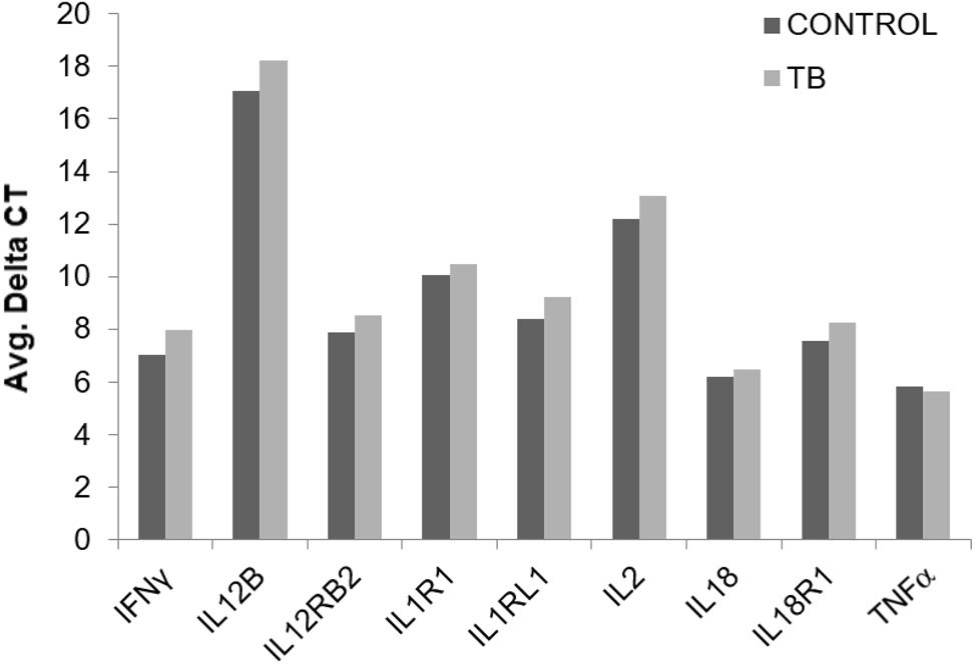
The bar chart represents the differences in the average delta CT for classical pro-inflammatory cytokines between TB patients and uninfected controls.

**Figure 5:**
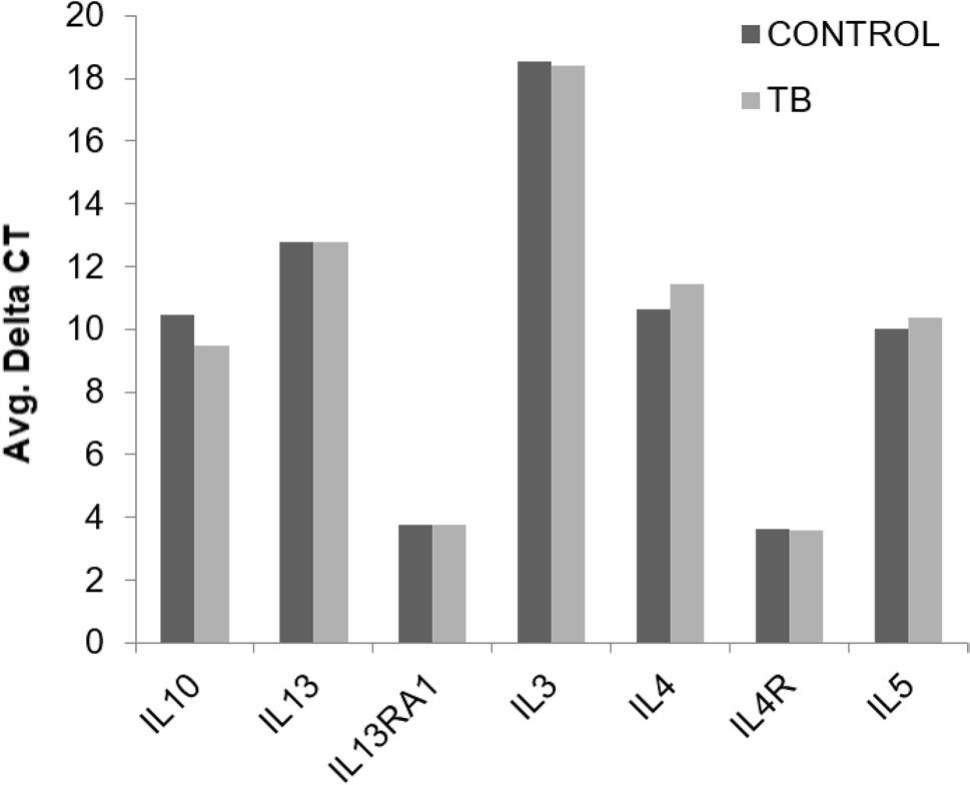
The bar chart represents the differences in the average delta CT for classical anti-inflammatory cytokines between TB patients and uninfected controls.

Our data indicate a significant upregulation of transcripts for IL-27, IL-15, TLR4, IRF1 and STAT1 in unstimulated PBMCs from active TB compared to uninfected individuals. However, no significant differences were observed in the classical pro-inflammatory cytokines such as IFNγ, IL-12B, IL-12RB2, IL-2, IL-18, IL-18R1, IL-1R1, IL-1RL1 and TNFα, suggesting a unique profile of immune activation in newly active TB compared to uninfected controls (Fig. 4). In agreement, our data identified that key transcripts such as IL-24, IL-2RA, CD80, CD28, PTGDR2, and NFATC1, essential for T-cell activation, proliferation and cellular differentiation were downregulated in active TB. In contrast, transcripts for IL-24, PTGDR2, NFATC1 and TGFβ have also been associated with an anti-inflammatory differentiation profile; however, none of the classical anti-inflammatory cytokines were significantly different between the uninfected and active TB population (Fig. 5). Further, TGFβ is a potent immune suppressive cytokine, but in our study, TGFβ was downregulated in active TB. *Our data differ from most of the data, including TB-infected individuals with a strong immune response, probably due to our cohort’s early stages of infection*^36,37^*. In addition, our data lack subsequent cellular activation or stimulation, the use of naïve PBMCs, providing an easy test to detect the early stages of TB in countries with limited access to clinical and molecular tools.*

### ROC (Receiver operating characteristic) curve analysis of upregulated significant differentially expressed genes (DEGs)

**Figure 6:**
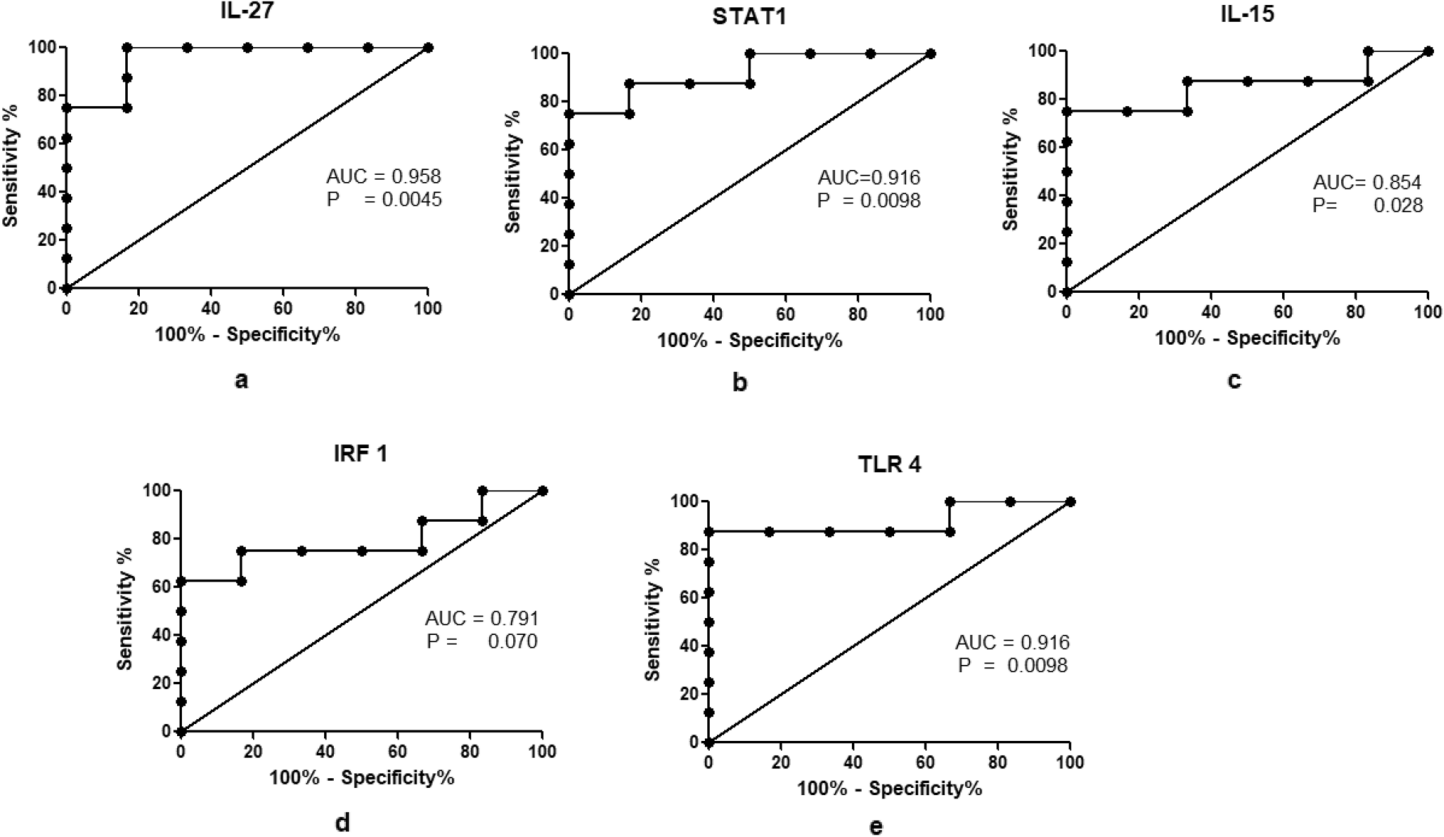
(**a**–**e**) ROC curve showing the accuracy of upregulated genes in discriminating between active TB and uninfected controls with AUC 0.79–0.958.

**Figure 7:**
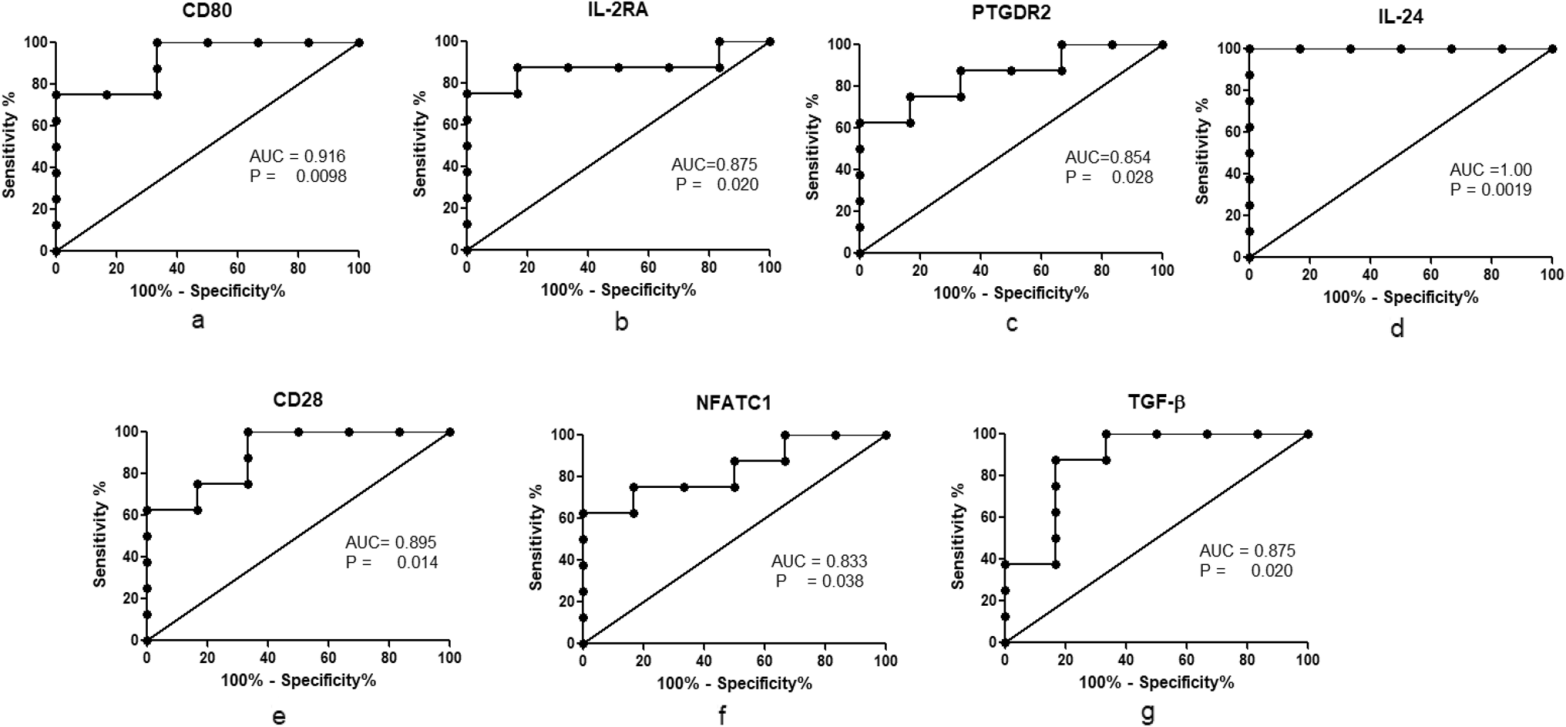
(**a**–**g**) ROC curve showing the accuracy of downregulated genes in discriminating between active TB and uninfected controls with AUC 0.833–1.00.

We performed a ROC analysis to assess the discriminatory power of identified significant DEGs. Among upregulated transcripts, the area under the curve (AUC) was determined to be 0.958 for IL-27, 0.916 for STAT1, 0.854 for IL-15, 0.791 for IRF1, and 0.916 for TLR4 (Fig. 6, Table 4). Whereas among downregulated transcripts, AUC was 0.895 for CD28, 0.875 for IL2RA, 0.854 for PTGDR2, 1.00 for IL-24, 0.916 for CD80, 0.833 for NFATC1and 0.875 for TGFβ (Fig. 7, Table 4). According to our results, the expression of IL-27, IL-24, STAT1, TLR4, and CD80 can be used to discriminate between active TB and uninfected controls, as the values of AUC are more than 0.9 for all 5 genes out of 12 significant DEGs (Table 4). The sensitivity and specificity at various cut off were also calculated for each candidate gene. At the highest likelihood ratio, the sensitivity and specificity for IL-27 were 100% and 83%; for IL-24, sensitivity of 100% and specificity of 83%; for CD80, sensitivity of 75% and specificity of 83%; for STAT1 and TLR4, the sensitivity of 87.5% and specificity of 83.8% respectively (Data is presented in supplementary Tables S1–S12).

**Table 4:** Summary of ROC analysis.

ROC				
Significant DEGs	Area	Std. Error	95% confidence interval	*P* value
IL-27	0.958	0.0514	0.8576 to 1.059	0.0045
STAT1	0.916	0.0758	0.7680 to 1.065	0.0098
IL-15	0.854	0.1093	0.6399 to 1.068	0.0282
IRF 1	0.791	0.1270	0.5427 to 1.041	0.0707
TLR 4	0.916	0.0846	0.7508 to 1.083	0.0098
CD 28	0.895	0.0862	0.7269 to 1.065	0.0142
IL-2RA	0.875	0.1040	0.6711 to 1.079	0.0201
PTGDR2	0.854	0.1028	0.6526 to 1.056	0.0282
IL-24	1.00	0.0000	1.000 to 1.000	0.0019
CD80	0.916	0.0761	0.7675 to 1.066	0.0098
NFATC1	0.833	0.1104	0.6169 to 1.050	0.0389
TGF-β	0.875	0.1055	0.6681 to 1.082	0.0201

#### Analysis of the interactions among DEGs identified in active TB patients

**Figure 8:**
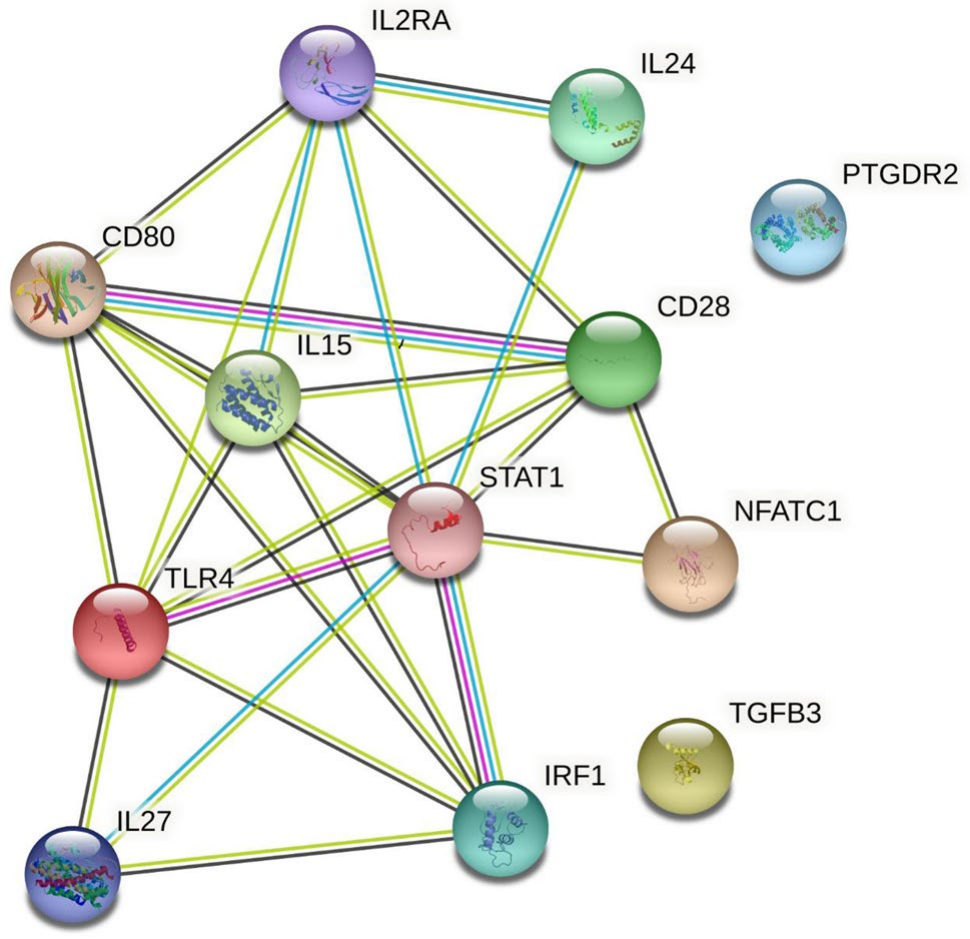
PPI interaction showed 12 nodes and 26 edges (*p* < 0.0001) between differentially expressed genes (DEGs).

The protein–protein interaction (PPI) network of differentially expressed genes (DEGs) comprised 12 nodes and 26 edges. The statistical significance of the PPI enrichment was found to be significant with a *p* value < 0.0001 (as depicted in Fig. 8). Most of the DEGs were found to have established interactions, primarily between IL-27, STAT1, IL-2, IFR1, IL-15, and IL-24, due to their crucial role in immune activation. However, the most distant genes were TGFβ and PTGDR2 according to PP1 interaction.

#### Functional annotation of differentially expressed genes (DEGs)

**Figure 9:**
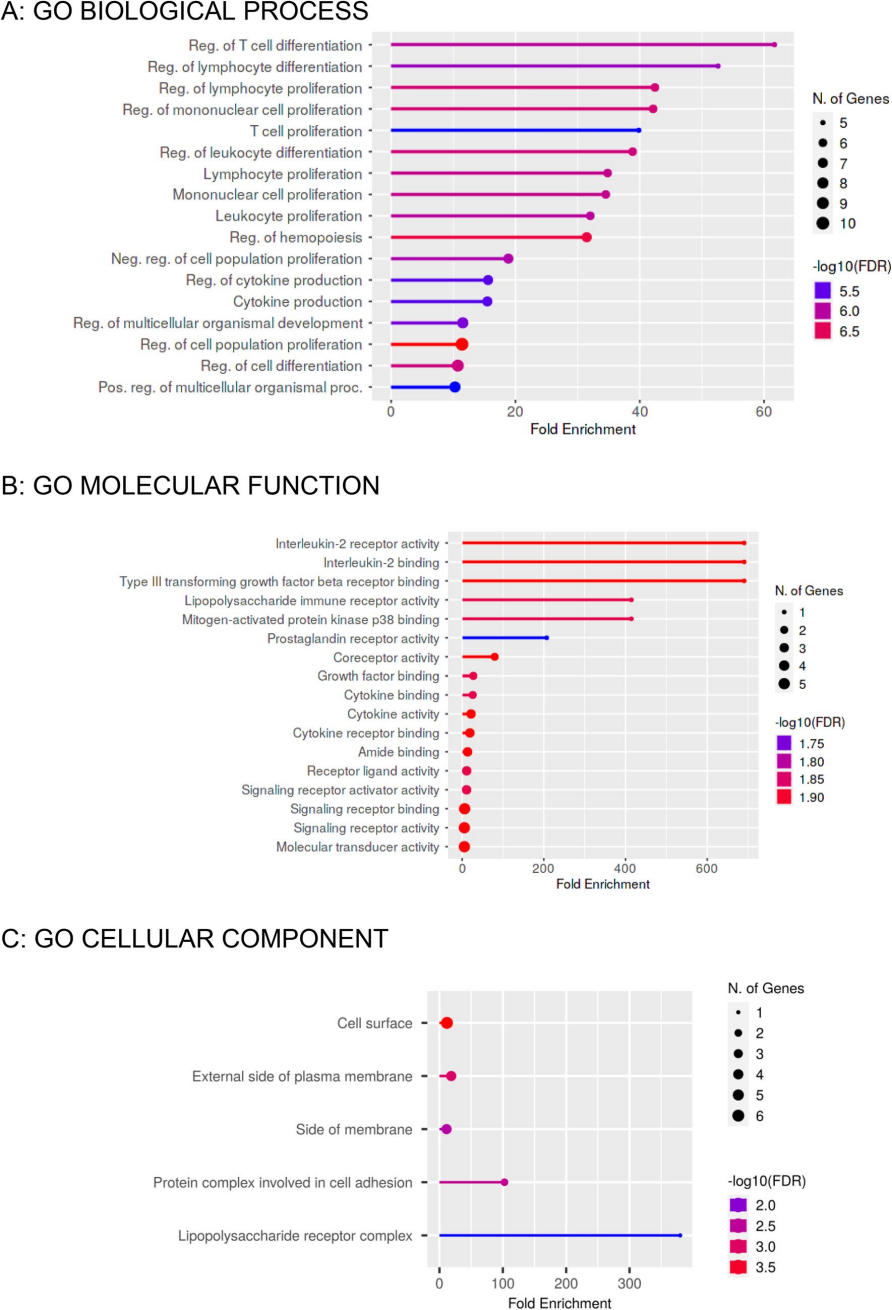
Gene Enrichment of the identified DEGs. Lollipop charts showing the top 17 GO terms for biological process (**A**), molecular function (**B**) and 5 GO terms for cellular component (**C**) ranked by fold enrichment.

Significant differentially expressed genes in active TB patients were further analyzed for functional annotation based on gene ontology classification according to biological process (BP), molecular function (MF) and cellular component (CC). According to BP enrichment analysis, majority of DEGs were associated with Reg. of cell differentiation (GO:0045580, GO:0045619, GO:1902105, GO:0045595), Reg. of cell proliferation (GO:0050670, GO:0032944, GO:0042127), Cell proliferation (GO:0042098, GO:0046651, GO:0032943, GO:0070661), Reg. of hemopoiesis (GO:1903706), Reg. of cytokine production (GO:0001817), Cytokine production (GO:0001816) as shown in Fig. 9A (Supplementary Table S13).

Moreover, DEGs at the molecular level (MF) were found to be involved in Interleukin-2 receptor activity (GO: 0004911), Interleukin-2 binding (GO:0019976), Type III transforming growth factor beta receptor binding (GO:0034714), Lipopolysaccharide immune receptor activity (GO:0001875), Mitogen-activated protein kinase p38 binding (GO:0048273), Prostaglandin receptor activity (GO:0004955), Co receptor activity (GO:0015026), Growth factor binding (GO:0019838), Cytokine binding (GO:0019955), Cytokine activity (GO:0005125), Cytokine receptor binding (GO:0005126), Amide binding (GO:0033218), Receptor ligand activity (GO:0048018), Signaling receptor activator activity (GO:0030546), Signaling receptor binding (GO:0005102), Signaling receptor activity (GO:0038023), Molecular transducer activity (GO:0060089) as shown Fig. 9B (Supplementary Table S14).

Cellular component enrichment analysis shows that DEGs belong to the gene product located at the Cell surface (GO:0009986), External side of the plasma membrane (GO:0009897), Protein complex involved in cell adhesion (GO:0098636), Lipopolysaccharide receptor complex (GO:0046696), Side of the membrane (GO:0098552) as shown in Fig. 9C (Supplementary Table S15).

## Discussion

Cell-mediated immune responses are essential in TB pathology. The balance between pro and anti-inflammatory events is significant in controlling TB disease; however, the associated immune profiles vary among different populations *due to the complexity of the disease*^13,41–46^*. TB also compromises the immune system resulting in associated comorbidities and no full protection against TB vaccines*^25,35,47–51^*. More importantly, the early detection of TB infection and immune compromise could provide the basis for early treatment.*

Our study analyzed unstimulated PBMCs of recently infected active pulmonary treatment naïve TB patients and uninfected controls to observe early transcriptional changes. According to our findings, most of the studied genes showed minimal to average expression patterns; however, few unique clusters of genes exhibited substantial differences in expression profiles between groups. Active TB patients showed significant (p < 0.05) differential expression for 12 genes compared to uninfected controls. The genes identified in our study correspond to the cytokines (IL-27, IL-15, IL-24, IL-2RA, TGFβ), downstream signaling mediators (STAT1), transcription factors (IRF1, NFATC1), innate immune response gene (TLR4), T-cell stimulatory molecules (CD28, CD80) and T -cell differentiation marker (PTGDR2).

*Several studies investigated the cytokine profile in naïve and stimulated PBMCs obtained from TB patients with or without treatment at different stages of the disease, and most of these studies point to specific Th1 or Th2 responses according to the population analyzed*^18,21,52^*.* Pro-inflammatory cytokines (IL-1β, IL-2, IL-12, IFNγ, and TNFα) are essential to combat TB disease. In contrast, the anti-inflammatory (IL-4, IL-13, and IL-10) profile is mostly associated with chronic TB^53^. We observed a unique pattern of cytokines expression in unstimulated PBMCs of active TB patients. We found no significant difference in the mRNA transcripts for IFNγ, IL-12B, IL-12RB2, IL-18, IL-18R1, IL-1R1, IL-1RL1, IL-2, TNFα, IL-10, IL-4, IL-13, IL-3, IL-6, IL-5, IL-7, IL-9, IL-25 compared to uninfected controls. The cytokine transcripts that showed significant differential expression in unstimulated PBMCs from active TB patients were IL-27, IL-15, IL-24 and TGFβ in our study.

We found significant upregulation in transcripts for STAT1, and IRF1, which are the essential transcription factors for cytokine signaling such as Jak-Stat pathway^54^. However, transcription factors and signaling mediators that are substantially involved in the differentiation and activation of pro/anti-inflammatory profiles were downregulated (TBX21, GATA3, NFATC1, NFATC2, STAT4, STAT6, IRF4, JAK1, CREBBP), and that corresponds to the reduced expression of classical inflammatory responses seen in our TB patients^55,56^. For chemokines and their receptors analyzed (CCL11, CCL5, CCL7, CCR2, CCR3, CCR4, CCR5, CXCR3), the findings were not significant in our study; however, most of the chemokine transcripts were downregulated in active TB compared to controls, which shows that essential chemokine responses to establish protective immunity were also diminished or absent in the early phase of TB infection.

We identified a unique signature of upregulated (IL-15, IL-27, STAT1, TLR4, IRF1) and downregulated (IL-2RA, IL-24, TGFβ, CD28, CD80, NFATC1, and PTGDR2) biomarkers from unstimulated PBMCs in the early phase of TB infection. We performed a ROC analysis to test the diagnostic performance of these biomarkers. In upregulated markers, IL-27, STAT1, and TLR4 showed the highest discriminatory power with AUC values 0.958 (*p* = 0.0045), 0.916 (*p* = 0.0098) and 0.916 (*p* = 0.0098), respectively. In contrast, among seven downregulated genes, IL-24 and CD80 showed the best discrimination between active TB and uninfected controls with AUC 1.00 (*p* = 0.0019) and 0.916 (*p* = 0.0098), respectively, in our study.

IL-27 is a heterodimeric immune modulatory cytokine. The signaling pathway of IL-27 involves STAT1 and IRF1 as important mediators. Several studies investigated the discriminatory potential of IL-27 in TB^57–63^. It is reported that increased expression of IL-27 was found in BAL fluid of pulmonary TB^64^. Similarly, IL-27 expression was a valuable indicator for diagnosing Tuberculous Pleural effusion (TPE)^61^. Our study was conducted on blood PBMCs of active pulmonary TB patients, and the only transcripts that showed significant upregulation up to 3.5 folds were mRNA for IL-27, so our findings support using IL-27 as a diagnostic biomarker for the *early stages* of pulmonary TB. *These findings provide potential biomarkers for early intervention and treatment.*

We also found higher values of AUC for STAT1 and TLR4; both are upregulated in active TB and can be used to discriminate between active TB and non-TB, according to our results. STAT1 has also been identified as a key gene and a potential biomarker in TB^65–67^, and our results also support this finding in pulmonary TB. Toll-like receptors play a significant role in Mtb recognition and to mount an initial response against Mtb^68^. Therefore, increased expression of TLR is not surprising, as we enrolled patients in the early stages after acquiring the disease. Similarly, IL-15 has been reported as discriminatory between latent and active TB^69^. We also found higher expression of IL-15 for active TB compared to uninfected controls; however, we didn’t analyze latent TB cases in our study. AUC for IL-15 is comparatively lower compared to other markers, so IL-15 might be a supplementary marker. Among downregulated transcripts, the discriminatory potential of IL-24 was best with AUC 1.00. According to the previous studies, IL-24 was decreased in patients with TB^70^. It is further reported that IL-24 stimulates IFNγ expression and could be a potential biomarker for TB^71^**.** We observed significantly decreased expression of IL-24 and no significant upregulation of IFNγ in unstimulated PBMCs of active TB patients. This aligns with previous observations, and our data support further investigations on IL-24 as a therapeutic biomarker.

A bioinformatics analysis of differentially expressed genes (DEGs) revealed strong interactions among certain immune-related genes, such as IL-27, STAT1, TLR4, and others, involved in pro-inflammatory responses. The analysis also showed that the most distant genes in the interaction network were TGFβ and PTGDR2. The gene ontology enrichment analysis revealed that most identified DEGs are associated with key biological processes like immune activation, cell proliferation and differentiation, and immune regulation. In terms of the molecular function, the DEGs were mainly enriched in cytokine activity and signaling pathways, and at the cellular level, the products of these genes were found to be cell surface proteins, external membrane components, and adhesion molecules. These findings are based on bioinformatics analysis, but further validation through translation profiling of the identified DEGs is necessary to determine their utility as biomarkers.

In our study on active pulmonary TB patients, the immune profile observed in unstimulated PBMCs was not based on classical cytokines. However, increased expression of IL-27, STAT1, IRF1, TLR4, and IL-15 may be key players in the early pathogenesis of TB. In addition, plasma concentrations of these markers might have different levels, but we didn’t assess plasma levels in our study. We also lacked a translation profile of gene signatures identified in active TB in our study. Therefore, translation studies are required to validate the current findings, and more such studies are required to strengthen the findings within Pakistan and across different countries. This might help to build global data on immune profiles in different ethnicities and would help to devise medical tests for  TB diagnosis at the early stages and treatment based on immunotherapies to avoid immune-related complications in TB patients.

## Conclusion

Our study identified unique gene signatures (IL-27, STAT1, IRF1, TLR4, IL-15, IL-2RA, IL-24, TGFβ, CD28, CD80, NFATC1, and PTGDR2) that discriminate active pulmonary TB from uninfected controls using unstimulated PBMCs. In addition, the translation profile of the identified transcripts needs to be investigated for functional characterization and validation of its utility as a biomarker of early TB infection stages.

## Materials and methods

### Ethical consideration

The study was approved by the Institute of Ethical Review Board (IRB, # 346/DUHS-12) of DUHS, whereas informed consent from each participant and their parents/local guardian was also taken to fulfill all the ethical requirements of our study. All the protocols involved in this study were performed according to the standard guidelines of WHO.

### Study participants

A total of fifty-one recently infected active pulmonary treatment naïve TB patients from Tuberculosis Hospital (DUHS, Karachi, Pakistan) and twenty-five healthy uninfected controls from different areas of Karachi were enrolled for this study. The recruitment was done based on physical examination, clinical symptoms, and laboratory assessments. *All patients were recruited within 4–6 weeks of the first clinical manifestations.* These patients were then referred to the counselor for risk assessment and demographic details to be recorded. Moreover, the BCG vaccine is mandatory for every Pakistan-born newborn as an integral part of the routine EPI program. Therefore, all participants were verified by BCG vaccination scar.

### Brief laboratory procedures for TB diagnosis

Patients with recent onset clinical TB symptoms, positive TST (> 5 mm is considered positive), as well as positive chest X-rays were further confirmed by sputum analyses using Acid-fast Bacilli (AFB) microscopy (ZN), culture (Bactec MGIT 960) and nucleic acid (GeneXpert) identification for Mtb at Tuberculosis laboratory. Moreover, TB patients underwent HIV testing as a part of the study protocol. Initially, HIV testing was performed through a rapid ICT method (Alere Determine™ HIV Combo), followed by ELISA (Genscreen ultra-HIV Ag-Ab-Bio-Rad Kit and Lab System Kit). All the protocols involved in this study were performed according to the standard guidelines of WHO.

### Selection of patients

Based on the above mentioned investigations, only those patients (n = 31 out of 51) under the age group of 15–55 years and categorized as Active Pulmonary TB patients by clinical experts were selected to collect blood samples. At the same time, individuals with long-term, secondary infection or history of Mtb infection, relapsed or extra-pulmonary TB, and individuals with co-morbidities such as HIV, HCV, and HBV infections and *other pulmonary diseases including asthma and pneumonia* were excluded (n = 20) from the study.

### Specimen collection and isolation of peripheral blood mononuclear cells (PBMCs)

Blood samples were collected from each participant (TB patients: n = 31, controls: n = 25) by venipuncture and stored in a heparin collection tube. All samples were processed within 15 min of the blood being drawn to isolate PBMCs. For this purpose, Ficoll Histopaque (Sigma, Aldrich) was used as a density gradient medium, which separates PBMCs from red blood cells and granulocytes via centrifugation. Briefly, 12 ml of blood was collected into heparinized tubes. Thenthe tube was centrifuged at 1200 r.p.m. speed for 12 min. to remove the platelet-rich plasma (PRP). DPBS (Dulbecco’s phosphate-buffered saline, Sigma) was added to the remaining portion of the blood to make up the original volume and mixed it well. Subsequently, blood sample was layered onto the histopaque tube (1:1) and centrifuged at 2000 r.p.m. for 20 min. A layer of mononuclear cells was aspirated carefully with the help of a fine, sterile pasture pipette and transferred into another sterile tube. Then aspirated layer was washed 2 to 3 times with DPBS buffer and centrifuged at 2600 r.p.m. for 10 min. at 4 °C. Lastly, the supernatant was carefully decanted. Then 1 ml of lysis buffer (Trizol) was added to the cell pellet and mixed it well to lyse the cells and finally, the samples were stored at − 80 °C till further process.

### RNA isolation

Cell lysate was processed for RNA extraction using an RNeasy Mini kit (Qiagen, Cat No. 74104). RNA was treated with DNase to remove contaminated genomic DNA. The quality of the RNA product was determined by an O.D. (optical density) 260/280 ratio ≥ 1.8 and OD 260/230 ratio ≥ 1.5 on a spectrophotometer, then gel (1.2%) electrophoresis was performed using 5 µl RNA volume. RNA was quantified using a spectrophotometer (Cadex, CITY) and Qubit RNA assay Kit (life technologies, CA). Then different RNA volume (1–5 µl) was used to ensure the uniformity in RNA amount (0.5 µg) for cDNA synthesis.

### cDNA preparation and PCR array

cDNA preparation was performed using RT2 first strand kit (Qiagen Cat No. 330401) according to the manufacturer’s instructions. We performed Polymerase Chain reactions to check cDNA synthesis using housekeeping genes as Primers. This study used commercially available beta globulin (B2M) primers (Cat no. 4333766F, Fisher Scientific). Further, cDNA samples were subjected to RT2 Profiler PCR Array (Human Th1-Th2, Catalog No. CAPH13039C, SA Biosciences). Each plate was implanted with eighty-four (n = 84) inflammatory genes such as chemokine and their receptors (CCL11, CCL5, CCL7, CCR2, CCR3, CCR4, CCR5, CXCR3), cytokine and their receptors (IFNγ, IL12B, IL12RB2, IL18, IL18R1, IL1R1, IL1RL1, IL2, IL2RA, IL10, IL13, IL13RA1, IL24, IL25, IL27RA, IL27, IL15, IL3, IL4, IL4R, IL5, IL6, IL6R, IL7, IL7R, IL9, GFI1), immune suppressive markers (TGFβ, SOCS1, SOCS5, PTPRC), TNF family (TNF, TNFRSF8, TNFRSF9, TNFSF4), cell surface markers (CD27, CD28, CD4, CD40LG, CD80, CD86), transcription factors (NFATC1, NFATC2, STAT1, STAT4, STAT6, TBX21, GATA3, CEBPB, CREBBP), signaling pathways (IRF1, IRF4, JAK1, JAK2, TLR4, TLR6, SPP1, LAT, MAPK8, TYK2), immune check point markers (ICOS, CTLA4, LAG3), growth factors (CSF2, VEGFA), apoptotic markers (MAF, BCL6, PCGF2, FASLG, LTA), other Immune transcripts and receptors (SFTPD, SLC11A1, HAVCR2, YY1, EBI3, PTGDR2). For quality control and normalization of the gene expression data, RT2 profiler assay is given with three internal controls (HGC, RTC, PPC) and five reference genes (ACTB, B2M, GAPDH, RPLP0& HPRT1) respectively (*Detailed description on NCBI version of the genes is available in Supplementary Table* S16)*.* Assays were performed on Real-time PCR (StepOne plus instrument—Applied Biosystems) according to the manufacturer’s instructions.

### Statistical analysis

The data was analyzed through Delta Delta CT (∆∆CT) method, where Delta CT (∆CT) was calculated between the gene of interest (GOI) and an average of housekeeping genes (HKG), followed by ∆∆CT calculations for fold change (FC). The fold change values were further transformed into a log_2_ base. Furthermore, the statistical significance (p value) was calculated using the student’s T-test with two-tailed distribution, unpaired and two-sample equal variance analysis. ROC analysis and graphical presentations were performed using Graph Pad Prism and online Qiagen software for data analysis.

#### Pathway analysis of significant differentially expressed genes (DEGs)

For functional annotation of DEGs, ShinyGO tool version 0.76 (http://bioinformatics.sdstate.edu/go/) was used. Fold enrichment analysis of significant differentially expressed genes was determined using gene ontology based on different categories such as biological process, molecular function, and cellular component to observe the association of identified DEGs with certain pathways at the molecular level and their location in a cell. We also investigated interactions of DEGs with the help of the String database, version 11.5 (https://string-db.org/cgi/network), to assess the biological networking among 12 DEGs that are significant from the obtained data set of 84 genes.

### Ethical approval and informed consent

Ethical approval was obtained from Civil Hospital Karachi & Dow University of Health Sciences to conduct this project IRB-346/DUHS-2012, and each participant was requested for written consent before enrolment for the study.

## Supplementary Information

Supplementary Tables.

Supplementary Tables.

Supplementary Tables.

## Data Availability

All data is available in the supplementary files with the manuscript.
