# Supplementary material for: Identification of immune biomarkers in recent active pulmonary tuberculosis
Source: Sci Rep. 2023 Jul 17;13:11481. doi: 10.1038/s41598-023-38372-7 (PMC10352308; doi:10.1038/s41598-023-38372-7)
Supplement: Supplementary file 2 — Supplementary Tables. [file 41598_2023_38372_MOESM2_ESM.docx]

**SUPPLEMENTARY TABLES**

Table S13: GO Biological Process

Table S14: GO Molecular Function

Table S15: GO Cellular Component
