# Supplementary material for: Identification of immune biomarkers in recent active pulmonary tuberculosis
Source: Sci Rep. 2023 Jul 17;13:11481. doi: 10.1038/s41598-023-38372-7 (PMC10352308; doi:10.1038/s41598-023-38372-7)
Supplement: Supplementary file 3 — Supplementary Tables. [file 41598_2023_38372_MOESM3_ESM.docx]

| **GenBank** | **Symbol** | **GenBank** | **Symbol** | **GenBank** | **Symbol** |
| --- | --- | --- | --- | --- | --- |
| NM_001706 | BCL6 | NM_002188 | IL13 | NM_002750 | MAPK8 |
| NM_002986 | CCL11 | NM_001560 | IL13RA1 | NM_172390 | NFATC1 |
| NM_002985 | CCL5 | NM_000585 | IL15 | NM_012340 | NFATC2 |
| NM_006273 | CCL7 | NM_001562 | IL18 | NM_007144 | PCGF2 |
| NM_001123396 | CCR2 | NM_003855 | IL18R1 | NM_002838 | PTPRC |
| NM_001837 | CCR3 | NM_000877 | IL1R1 | NM_003019 | SFTPD |
| NM_005508 | CCR4 | NM_016232 | IL1RL1 | NM_000578 | SLC11A1 |
| NM_000579 | CCR5 | NM_000586 | IL2 | NM_003745 | SOCS1 |
| NM_001242 | CD27 | NM_006850 | IL24 | NM_144949 | SOCS5 |
| NM_006139 | CD28 | NM_022789 | IL25 | NM_000582 | SPP1 |
| NM_000616 | CD4 | NM_145659 | IL27 | NM_007315 | STAT1 |
| NM_000074 | CD40LG | NM_004843 | IL27RA | NM_003151 | STAT4 |
| NM_005191 | CD80 | NM_000417 | IL2RA | NM_003153 | STAT6 |
| NM_006889 | CD86 | NM_000588 | IL3 | NM_013351 | TBX21 |
| NM_005194 | CEBPB | NM_000589 | IL4 | NM_003239 | TGFB3 |
| NM_004380 | CREBBP | NM_000418 | IL4R | NM_138554 | TLR4 |
| NM_000758 | CSF2 | NM_000879 | IL5 | NM_006068 | TLR6 |
| NM_005214 | CTLA4 | NM_000600 | IL6 | NM_000594 | TNF |
| NM_001504 | CXCR3 | NM_000565 | IL6R | NM_001243 | TNFRSF8 |
| NM_005755 | EBI3 | NM_000880 | IL7 | NM_001561 | TNFRSF9 |
| NM_000639 | FASLG | NM_002185 | IL7R | NM_003326 | TNFSF4 |
| NM_002051 | GATA3 | NM_000590 | IL9 | NM_003331 | TYK2 |
| NM_005263 | GFI1 | NM_002198 | IRF1 | NM_003376 | VEGFA |
| NM_004778 | PTGDR2 | NM_002460 | IRF4 | NM_003403 | YY1 |
| NM_032782 | HAVCR2 | NM_002227 | JAK1 | NM_001101 | ACTB |
| NM_012092 | ICOS | NM_004972 | JAK2 | NM_004048 | B2M |
| NM_000619 | IFNG | NM_002286 | LAG3 | NM_002046 | GAPDH |
| NM_000572 | IL10 | NM_014387 | LAT | NM_000194 | HPRT1 |
| NM_002187 | IL12B | NM_000595 | LTA | NM_001002 | RPLP0 |
| NM_001559 | IL12RB2 | NM_005360 | MAF | - | - |

Supplementary Table S16: NCBI Version of analyzed genes
